# Evolution of Coronary Calcium Screening for Assessment of Atherosclerotic Cardiovascular Disease Risk and Role in Preventive Cardiology

**DOI:** 10.1007/s11883-022-01073-z

**Published:** 2022-11-14

**Authors:** Nathan D. Wong

**Affiliations:** Heart Disease Prevention Program, Division of Cardiology, C240 Medical Sciences, University of California, Irvine, CA 92697 USA

**Keywords:** Coronary calcium, Cardiovascular disease, Atherosclerosis, Risk factors, Epidemiology

## Abstract

**Purpose of Review:**

Coronary artery calcium (CAC) is an important measure of subclinical atherosclerosis and strongly predicts atherosclerotic cardiovascular disease (ASCVD) outcomes. The purpose of this review is to discuss the key studies that have helped to establish its role as an important screening tool and its place in preventive cardiology.

**Recent Findings:**

Epidemiologic studies document a strong relation of age, race/ethnicity, and risk factors with the prevalence and extent of CAC. Large-scale registry and prospective investigations show CAC to be the strongest subclinical disease predictor of ASCVD outcomes, with higher CAC scores associated with successively higher risks and those with a CAC score of 0 having a long-term “warranty” against having events. Moreover, CAC is associated with greater initiation of preventive health behaviors and therapy. Current US guidelines utilize CAC to inform the treatment decision for statin therapy. Further study is underway to document whether CAC screening will ultimately improve clinical outcomes.

**Summary:**

CAC is well established as the most important subclinical cardiovascular disease measure for prediction of future ASCVD outcomes and can be used for informing the treatment decision for preventive therapies.

## Introduction

With atherosclerotic cardiovascular diseases (ASCVD) the leading causes of death globally, there has been the search for newer modalities to detect ASCVD earlier, in its subclinical phase, before clinical events occur, which in a significant number of people are initially fatal. It is well established that some two-thirds of myocardial infarctions are the result of coronary stenoses of less than 50%, with only one-seventh of myocardial infarctions occurring as a result of blockages exceeding 70% [1]. It has also been previously noted that the majority of people destined to die suddenly will not have a positive exercise test, the likely reason of their demise being a mild, non-flow limiting coronary plaque present before the development of an occlusive thrombus [2].

While current guidelines continue to focus on estimation of global (e.g., 10 year) risk of ASCVD [3••] as the starting point in evaluation of ASCVD risk for the consideration of preventive therapies, it has long been noted that despite such office-based risk assessment approaches, there remains a substantial gap in the detection of asymptomatic persons who ultimately develop coronary heart disease and that risk scores (including the previous Framingham and European risk scores) emphasizing the use of classic risk factors are only moderately accurate for the prediction of short- and long-term risk of manifesting a major coronary event [4]. Moreover, it has been reported that less than a third of persons with coronary heart disease have two or more traditional risk factors [5]. This has motivated the need for identifying newer screening tests for atherosclerosis. The 34^th^ Bethesda Conference of the American College of Cardiology [6] noted that a good screening test needs to provide (1) an accurate determination of the likelihood that an asymptomatic person has the condition of interest (e.g., coronary artery disease) (accuracy), (2) needs to be reproducible (reliability), (3) detect individuals where early intervention is likely to have a beneficial impact, and (4) should provide incremental value to risk predicted by office-based risk assessment.

A variety of tools to screen for atherosclerosis have been proposed and/or studied over the past several decades, including carotid ultrasonography, coronary computed tomography, magnetic resonance imaging, ankle brachial index, brachial ultrasound, and radial and fingertip tonometry. Much of the research has focused on carotid ultrasound in the measurement of carotid intimal medial thickness along with carotid plaques, ankle brachial index for identifying peripheral arterial disease, and coronary computed tomography for the assessment of coronary artery calcium. This review will focus on the establishment of coronary calcium screening by computed tomography as a tool for assessing ASCVD risk and its role in preventive cardiology.

## Early Development of Coronary Calcium Screening

More than 60 years ago, Blankenhorn and Stern described the significance of calcification of the coronary arteries as a marker of atherosclerosis [7]. Post-mortem studies documented the extent of coronary artery calcium (CAC) to relate to severity of coronary stenosis and frequency of myocardial infarctions [8, 9]. Studies examining CAC utilizing cardiac fluoroscopy going back nearly 50 years ago documented calcium to be invariably an indication of intimal atherosclerosis [10] and to relate to obstructive coronary disease [11, 12]. While studies involving fluoroscopy at best provided semi-quantification of coronary calcification, Agatston and colleagues in 1990 [13] published the first quantitative assessment of CAC utilizing ultrafast computed tomography (CT), creating a total calcium score based on the number, areas, and peak Hounsfield CT numbers of the calcium lesions detected, a score still in use today clinically and in research. They described the age-related increase in calcium score as well as its powerful sensitivity and specificity for the detection of angiographically documented coronary disease. At the time of that publication, the authors noted that the detection of CAC “may” have prognostic significance and that “further studies using this new diagnostic tool to determine the prognostic value of precise coronary calcium quantification are in progress”.

## Early Epidemiologic Studies of Coronary Calcium

The South Bay Heartwatch was the first National Institutes of Health funded protocol to examine the prognostic significance of CAC measured initially by cardiac fluoroscopy and then by electron beam or ultrafast CT. In this study of 1461 higher risk asymptomatic subjects, we showed the prevalence of CAC to increase from < 20% in persons aged 40–49 years to > 80% in those aged 80 years or greater, and the presence of CAC to be associated with a 1.4-fold greater risk of coronary heart disease events after 1 year after adjustment for age, sex, and risk factors [14]. This study was also instrumental in first documenting significant ethnic differences in CAC, with African-American race/ethnicity being associated with a 31% lower likelihood of having CAC in age, sex, and risk factor-adjusted analyses [15]. The South Bay Heartwatch also first showed CAC scores to provide incremental prognostic value for the composite of coronary death or nonfatal myocardial infarction over global risk assessment (using the Framingham risk score [FRS]), showing an improvement in the c-statistic from 0.63 from FRS alone to 0.68 for FRS plus CAC (*p* < 0.001 for comparison) [16].

Other early studies, typically in self or physician-referred subjects for CAC screening also demonstrated strong relationships with age, risk factors and cardiovascular outcomes with electron beam or ultrafast CT. We showed CAC prevalence to increase dramatically with age from 15% in men and 30% in women under the age of 40 to 93% in men and 75% in women aged 70 years and over and in multiple logistic regression, age, male gender, hypertension, diabetes, hypercholesterolemia, and obesity to be independently associated with CAC [17]. Moreover, in a cohort of 881 subjects with mean follow-up of 3.3 years for cardiovascular events, we showed from Cox regression models, adjusted for age, gender, and coronary risk factors, the relative risks (*RR*) for those with CAC scores of 81 to 270 of 4.5 (*p* < 0.05) and 271 or greater of 8.8 (*p* < 0.001) [18]. Subsequently, in a much larger cohort of 5,635 low to intermediate risk persons followed for a mean of 37 months, Kondos and colleagues [19] showed cardiac events to be more strongly related to presence of CAC in men (*RR* = 10.5, *p* < 0.001) than in women (*RR* = 2.6, *p* = 0.037) beyond the contribution of risk factors. Shaw et al. in the same year showed the strong prognostic significance of CAC scores (ranging up to 1000 and more) in more than 10,000 men and women with risk factors for all-cause mortality over follow-up extending beyond 4 years [20]. Budoff and colleagues further documented the strong prognostic significance of a wide range of CAC scores among 25,253 patients followed for more than 10 years [21]. This study showed 2.2-, 4.5-, 6.4-, 9.2-, 10.4-, and 12.5-fold greater adjusted risks of all-cause mortality for scores of 11 to 100, 101 to 299, 300 to 399, 400 to 699, 700 to 999, and > 1,000, respectively (*p* < 0.0001), when compared with 0 with 10-year survival being 99.4% for a CAC score of 0 worsening to 87.8% for a score of > 1,000 (*p* < 0.0001).

## Lessons from the Multiethnic Study of Atherosclerosis (MESA) and Beyond

While most of the data described above regarding the prognostic significance of CAC relied on self and physician-referred cohorts, there was a need to document the value of CAC in larger population-based studies not subject to the potential self-selection issues of prior studies. A major premise of the Multiethnic Study of Atherosclerosis (MESA) (initially known as the Subclinical Cardiovascular Disease Study in the application phase) was to document in a largely population-based cohort the prognostic significance of CAC and its value in relation to standard risk factors and other measures of subclinical atherosclerosis in a multiethnic cohort which not only included African-American participants, but for the first time large numbers of Hispanic and Asian participants. MESA and the Coronary Artery Risk Development in Young Adults (CARDIA) studies were the first multicenter NIH studies to include coronary calcium and included a centralized reading center. They not only examined CAC by electron beam CT, but also by then newer multidetector CT methods [22]. MESA demonstrated equivalent reproducibility and concordance for the detection of CAC between these two different types of scanners [23].

**Fig. 1:**
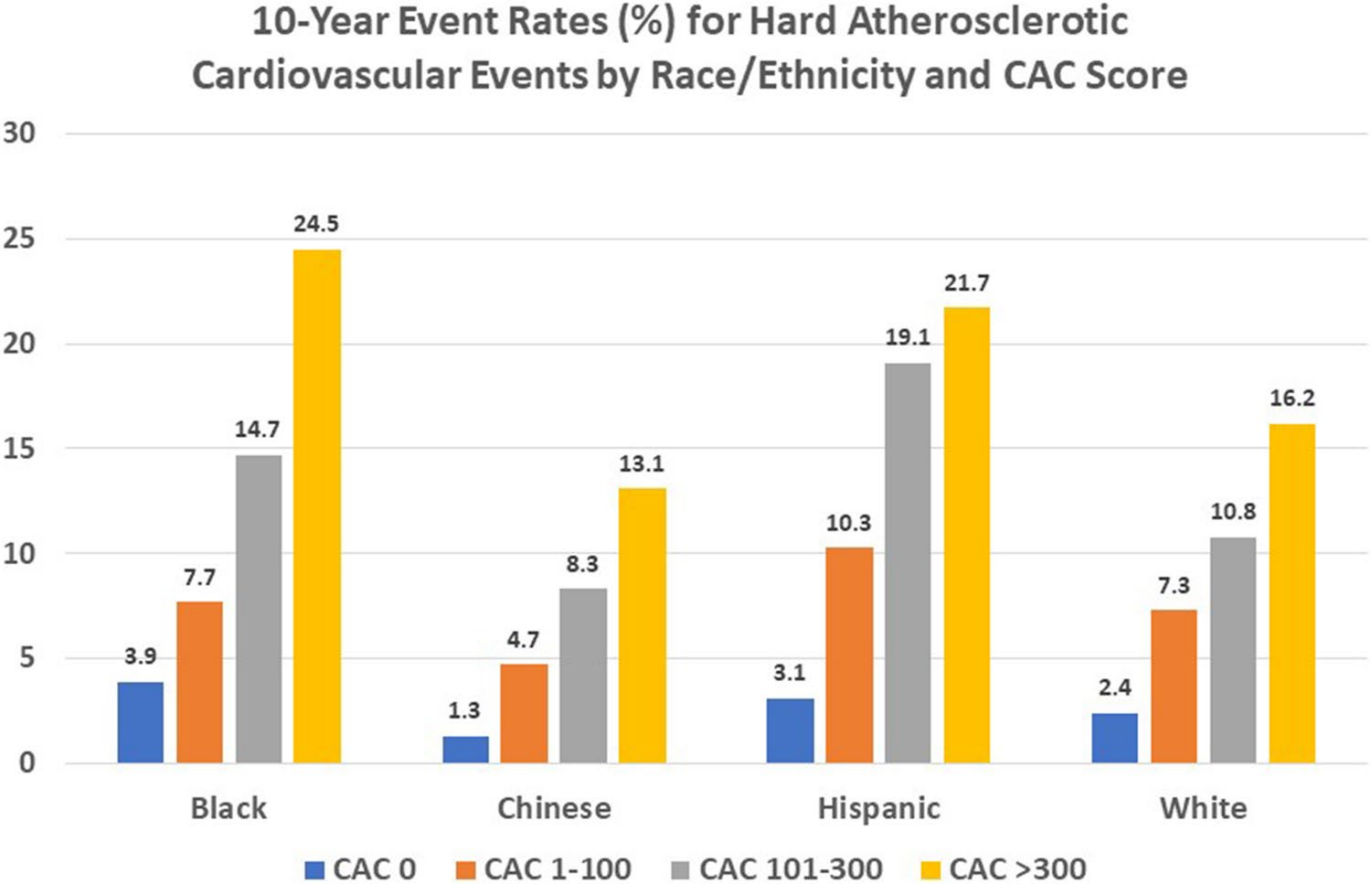
Ten-year rates (%) of hard atherosclerotic cardiovascular disease events in the multiethnic study of atherosclerosis by race/ethnicity and coronary artery calcium (CAC) score (data adapted from Budoff et al. [27••].)

Among the 6,814 participants scanned at the baseline exam in MESA, Bild et al. [24] described the ethnic distribution of CAC showing that compared to white participants, the relative risks of having CAC were 22%, 15%, and 8% lower in black, Hispanic, and Chinese participants. A more recent paper in collaboration with the MASALA (Mediators of Atherosclerosis in South Asians Living in America) study showed similar age-related trajectories in CAC prevalence among white and South Asian persons that was greater than other race/ethnic groups studied in MESA [25]. The seminal paper in MESA that first examined CAC in relation to ASCVD outcomes was published in 2008 by Detrano and colleagues [26] after a median follow-up of 3.8 years and overall showed the adjusted risk of a coronary event to be 7.7-fold greater among participants with CAC scores of 101–300 and 9.7-fold greater in those with scores above 300 (*p* < 0.001 for both comparisons) compared to 0. Importantly, it was the first paper to show consistency in risk prediction across four major ethnic groups with an improvement in the c-statistic beyond risk factors. More recently, Budoff and colleagues [27••] showed the continued prognostic value of CAC from 10 years of follow-up in MESA, with 10-year ASCVD event rates by CAC score ranging from 1.3% to 24.5% across different age, gender, and racial subgroups and noting that all participants with CAC > 100 regardless of demographic subset had 10-year ASCVD risks of > 7.5% (Fig. 1), indicating eligibility for a statin therapy according to guidelines. In addition, there was a 14% increase in risk for every doubling of CAC score.

The ability of CAC to improve risk prediction and reclassification beyond traditional risk factors is an important consideration for its utility as a screening tool. Besides the earlier reports described above from the South Bay Heart Watch [16] and MESA [26], Polonsky and colleagues [28] examined the ability of CAC to improve risk reclassification and found the addition of CAC to a model of age, sex, and risk factors resulted in a 25% net reclassification improvement (NRI); an additional 23% of those who experienced events were reclassified as high risk, and an additional 13% without events were reclassified as low risk when CAC was added. Moreover, Yeboah et al. [29] showed the addition of CAC to Framingham risk score improved the c-statistic far more (from 0.623 to 0.784, *p* < 0.001) than that provided by carotid intimal medial thickness, flow mediated dilatation, c-reactive protein, family history, or ankle brachial index, providing support to the American College of Cardiology Foundation (ACCF) / American Heart Association (AHA) 2013 risk assessment guideline [30] noting that “assessing CAC is likely to be the most useful of the current approaches to improving risk assessment among individuals found to be at intermediate risk after formal risk assessment”. Moreover, MESA investigators created a risk score with CAC incorporated, which further refined our ability to predict 10-year CHD event risk along with standard risk factors [31].

In young adults, the Coronary Artery Risk Development in Young Adults (CARDIA) study studied 3,043 adults who were scanned at ages 32–46 and followed for 12.5 years. Carr and colleagues [32] recently showed those with any CAC to have a fivefold greater risk of CHD events and a threefold greater risk of CVD events and within CAC score strata of 1–19, 20–99, and 100 and greater, the HR’s for CHD events were 2.6 (95% CI 1.0–5.7), 5.8 (95% CI 2.6–12.1), and 9.8 (95% CI 4.5–20.5). Most recently, Javaid and colleagues [33•] harmonized 3 major datasets: CARDIA, the Walter Reed Cohort and the CAC Consortium comprising 19,725 black and white individuals aged 30–45 and noted CAC > 0 to be 26% among White males, 16% among Black males, 10% among White females, and 7% among Black females. Moreover, a positive CAC scan automatically placed all females at > 90th percentile, White males at the 90th percentile at age 34 years, and Black males at age 37 years. They also created an interactive webpage allowing one to enter an age, sex, race, and CAC score to obtain the corresponding estimated CAC percentile.

MESA also has the most extensive data examining incidence and progression of CAC as a means of following changes in atherosclerosis. An initial paper [34] described rates of progression of CAC over 2.4 years, noting the incidence of newly detectable CAC averaged 6.6% per year with incidence increased steadily across age and median annual progression of CAC in those with existing CAC was 14 units in women and 21 units in men. Moreover, age, male gender, white race/ethnicity, hypertension, body mass index, diabetes mellitus, glucose, and family history of heart attack were associated with incidence and progression of CAC. We further examined the prognostic significance of progression of CAC in 5,682 adults in MESA with repeat CAC scans after a mean of 2.5 years with median follow-up for CHD events over 7.7 years; the cumulative incidence of CHD ranged from < 10% in those with CAC progression of < 100 units annually to over 30% in those with annual CAC score increases exceeding 300 [35]. We also demonstrated a greater incidence and progression of CAC in persons with metabolic syndrome or diabetes relative to those with neither of these conditions, as well as progression of CAC in these groups to be associated with a greater incidence of CHD events [36]. Moreover, in a more recent paper in collaboration with the MASALA study, Chinese, Black, and Latino men had significantly less CAC change compared to South Asian men, but no differences between South Asian and white men and there were no differences in CAC incidence or progression between South Asian women and women of other race/ethnic groups in MESA [37].

## Coronary Calcium and Cardiovascular Risk Assessment in Diabetes

**Fig. 2:**
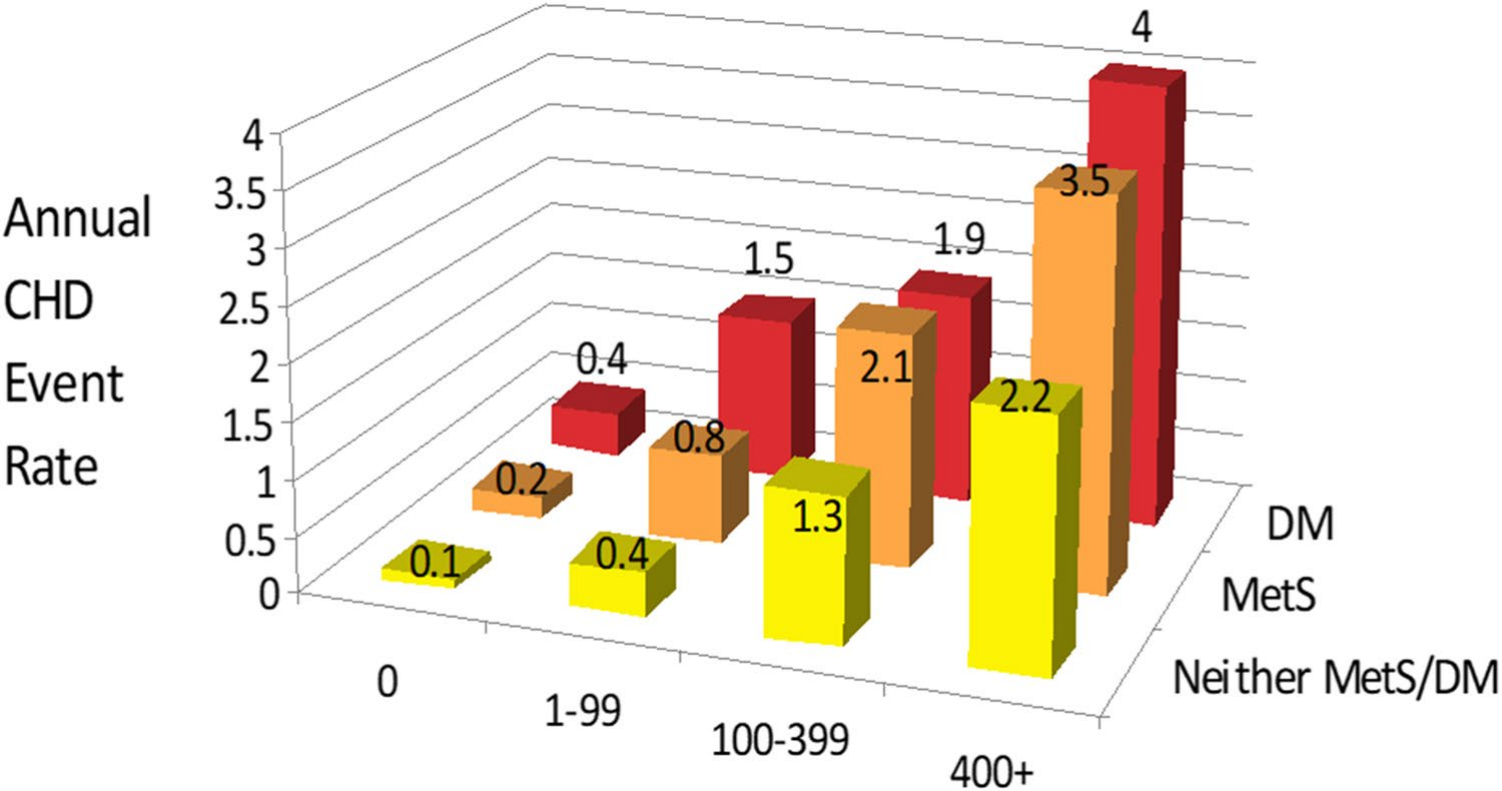
Annual coronary heart disease event rates (per 1000 person years) by coronary calcium score in persons with diabetes (DM), metabolic syndrome (MetS), or neither condition: multiethnic study of atherosclerosis (adapted from Malik et al. [42].)

While diabetes mellitus was considered in previous guidelines as a CHD risk equivalent, it is realized this is no longer the case. A large meta-analysis shows those with diabetes but no prior myocardial infarction to be at overall 44% lower risk of future CHD events than those with a prior myocardial infarction but no diabetes [38]. Also, in examining global risk in US adults with diabetes, we note a third of men and nearly half of women to be low or intermediate 10-year risk of CVD [39]. Several studies document the greater prevalence and extent of CAC as well as the prognostic value of CAC in those with diabetes. We showed in 1823 patients screened for CAC that the prevalence of CAC clearly increased from the reference group to those with metabolic syndrome or diabetes, with 53.5%, 58.8%, and 75.3%, respectively, among men and 37.6%, 50.8%, and 52.6%, respectively, among women. CAC prevalence was also greater with the number of components of the metabolic syndrome (0 to 5) from 34 to 58% [40]. Hoff et al. [41] also showed in a larger cohort of 30,904 asymptomatic individuals stratified by their self-reported diabetes status higher CAC scores in those with versus without diabetes; CAC score in the highest age/gender quartile was 70% greater for those with vs. without diabetes. Moreover, among those with metabolic syndrome or diabetes, we showed in MESA annualized CHD event rates vary by approximately tenfold ranging from CAC scores of 0 to 400 or greater, again confirming the wide heterogeneity in risk that exists in persons with these conditions [42] (Fig. 2). A further investigation of this cohort with longer-term follow-up for ASCVD events continues to document the value of CAC (along with duration of DM) for stratification of risk in persons with diabetes [43]. In a larger investigation from the Coronary Calcium Consortium [44•] comprising over 4,000 adults with diabetes, we showed CAC scores to be a stronger predictor for cardiovascular and total mortality in women compared to men with diabetes, possibly in part helping to explain previous reports showing diabetes to be a stronger predictor of CVD in women compared to men. Moreover, MESA, in collaboration with the Heinz Nixdorf Recall Study, studied 1,343 persons with diabetes with 8-year follow-up and created a risk score for incident CHD events that included age, sex, systolic blood pressure, duration of diabetes, and CAC scores and found this to improve the c-statistic for prediction of CHD events beyond that provided by the Framingham and United Kingdom Prospective Diabetes Study risk scores [45].

## Does Coronary Calcium Screening Improve Behavior, Treatment, and Outcomes?

CAC screening is an excellent example of the concept of “a picture is worth a thousand words.” We were among the first to demonstrate this concept more than 25 years ago with CAC scanning in an observational study of 703 men and women aged 28–84 where the extent of CAC was independently associated with self-report 1 to 2 years later of initiation of preventive behaviors; age, gender, and risk factor adjusted logistic regression showed log of CAC score to be associated with new aspirin usage, new cholesterol medication, consulting with a physician, losing weight, and decreasing dietary fat (all *p* < 0.01), but also increased worry (*p* < 0.001) [46]. In a subsequent study, Orakzai and colleagues [47] showed the greater the calcium score, the greater the likelihood of beginning preventive therapies; in multivariable analysis, greater baseline CAC was strongly associated with initiation of ASA therapy, dietary changes, and increased exercise. Moreover, Taylor and colleagues [48] showed in the Prospective Army Coronary Calcium (PACC) study the presence of CAC to be associated with greater incidence of statin and aspirin use over 6 years of follow-up. A more recently published meta-analysis pooling 6 studies involving 11,256 subjects noted aspirin, lipid-lowering medication, blood pressure-lowering medication initiation, increase in exercise, and dietary change to be greater in those with CAC > 0 versus CAC = 0 [49].

But whether randomization to calcium scanning (compared to no scanning) offers clinical benefit is still an open question. In the Early Identification of Subclinical Atherosclerosis by Noninvasive Imaging Research (EISNER), Rozanski and colleagues [50] showed 2:1 randomization of 2,137 middle-aged persons with risk factors to scanning versus no scanning after 4 years of follow-up to result in halting the change in Framingham Risk Score in the scan group compared to the no scan group (0.002 vs. 0.7, *p* = 0.003), with the baseline score being associated with improvement in risk factors; those with CAC > 400 vs. 0 had greater reductions in LDL-C, systolic blood pressure and were more likely to start new lipid, blood pressure, and aspirin therapy. Moreover, procedure and medication costs were 37% and 26% lower in the scan group compared to the no scan group. More recently, the DANCAVAS cardiovascular screening trial in 46,611 Danish men aged 65–74 years [51••] showed randomization to screening with CAC scanning, carotid ultrasound, and laboratory tests versus no screening showed after 5.6 years of follow-up a non-significant (*p* = 0.06) 5% reduction in the risk of total mortality (although a significant 11% reduction in risk in the subgroup < 70 years old), 9% reduction in risk of myocardial infarction, and 7% reduction in stroke. The ongoing Dutch Risk Or Benefit IN Screening for CArdiovascular disease (ROBINSCA) trial is currently investigating whether CAC screening followed by preventive treatment is effective in reducing CHD-related morbidity and mortality in asymptomatic individuals. A total of 43,447 potentially high-risk women and men from the national population registry have been randomly allocated (1:1:1) to either the control arm, intervention arm A (screening according to traditional risk factors using the SCORE algorithm) or intervention arm B (CAC screening). Differences in 5-year ASCVD event rates will be examined [52]. Those with greater 10-year SCORE risk or CAC were assigned to guideline directed medical therapy for primary or secondary prevention, respectively.

## Coronary Calcium Screening and the Guidelines: Implications for Preventive Cardiology

While CAC screening has been described by prior guidelines (2013) as useful for risk assessment in intermediate risk individuals, its role in the treatment decision has only been incorporated recently in guidelines [3••]. Essential evidence to help support its inclusion in the recent guidelines derives from a report from Nasir and colleagues [53] who examined from follow-up of MESA participants for ASCVD events stratified by CAC score and ASCVD risk group as calculated by the ASCVD pooled cohort risk calculator. While the 2013 Cholesterol Management guideline noted the statin benefit threshold to be at >  = 7.5% 10-year ASCVD risk, Nasir and colleagues showed that unless the 10-year risk was > 20%, those with a 0 calcium score had an estimated 10-year ASCVD risk (extrapolated from the existing follow-up in MESA at the time of the publication) below the 7.5% threshold for benefit (and number needed to treat of 64); however, those whose CAC scores were 100 or greater, regardless of 10-year risk level ≥ 7.5%, had ASCVD event rates estimated to be at or above this level (with a number needed to treat of 22), thus indicating a definite benefit from statin use. This report showed that of those eligible for statins, 44% actually had CAC = 0 at baseline and an observed 10-year ASCVD event rate of 4.2 per 1000 person years, suggesting they were below the threshold for benefit from statins. In a subsequent MESA report, Mitchell and colleagues [54] noted that those with CAC >  = 100 derive greater benefit from statin treatment (with number needed to treat of 12) where the incidence of major adverse cardiovascular events was much lower for those on statins vs. not on statins; however, in those with CAC < 100, there was little benefit seen. The long-term warranty against mortality or ASCVD events associated with a 0 CAC score has been well-documented by others, even showing its superior predictive value to low 10-year calculated risk or not having risk factors [55] and irrespective of number of risk factors [56], age [57], or LDL-C levels [58].

**Fig. 3:**
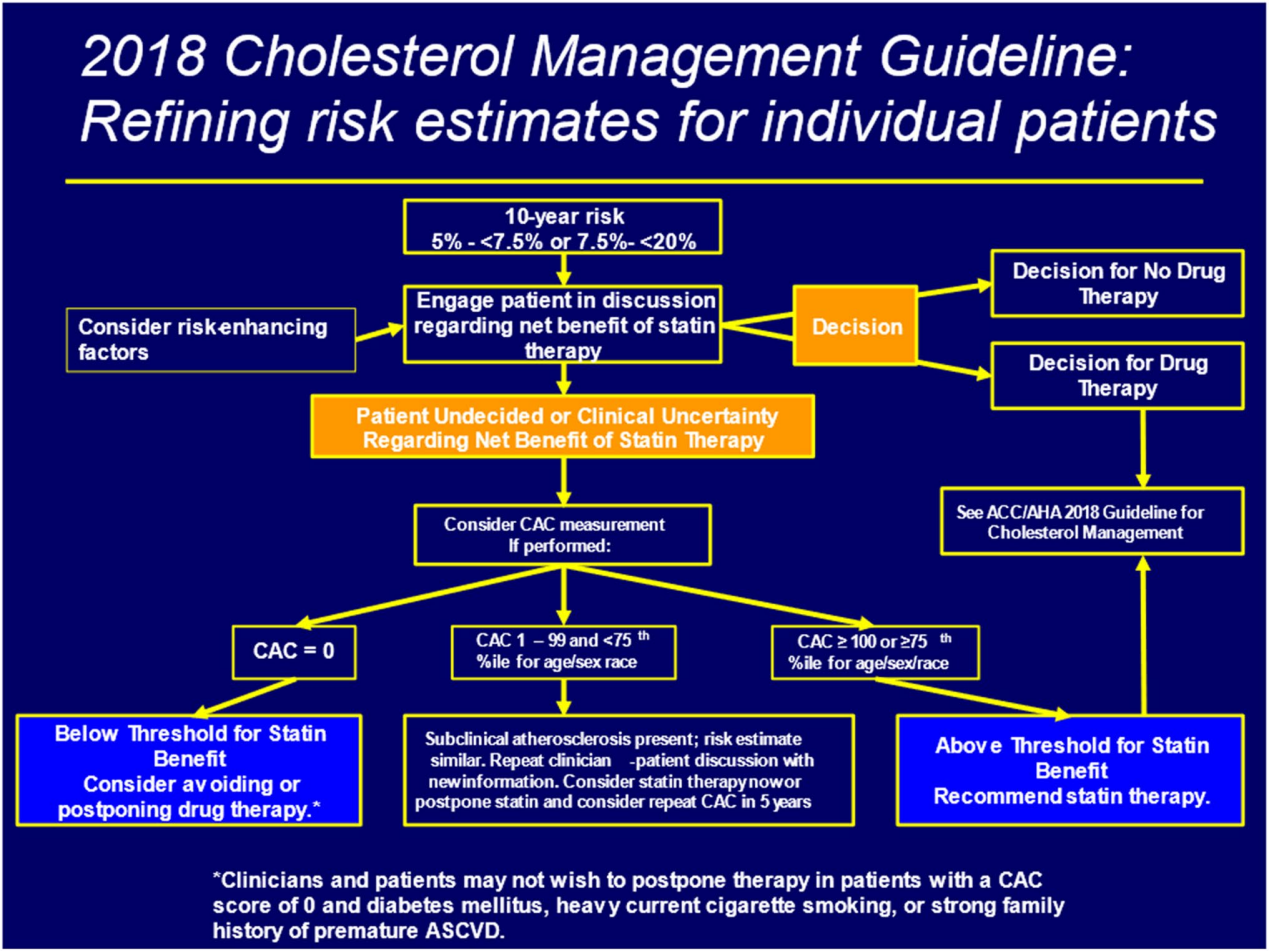
Role of coronary calcium scoring in the 2018 cholesterol management guideline (from Grundy et al. [3••].)

The 2018 ACC/AHA Multisociety Guideline for Cholesterol Management recommends consideration of CAC scanning where the treatment decision is still uncertain after global risk assessment (with the ASCVD pooled cohort risk estimator) and consideration of risk enhancing factors [3••] (Fig. 3). In those found to have a CAC score of ≥ 100 or ≥ 75^th^ percentile for age, sex, and ethnicity, there is a definite indication for statin therapy, preferably to lower LDL-C at least 50% (e.g., high intensity statin). In those with a CAC score of 0, withholding or delaying statin use is a consideration, except in those with diabetes, heavy cigarette smoking, or a strong family history of premature ASCVD where presumably the lifetime risk of ASCVD is high. Among those with CAC scores of 1–99 or < 75^th^%tile, there is the option to either consider statin therapy now or to postpone therapy and consider repeating the CAC scan in 5 years.

While similar society recommendations do not exist for other therapies at present, Miedema et al. [59] also show those without CAC to derive little benefit from aspirin therapy (with numbers needed to treat of > 500 whether or not 10-year ASCVD risk is >  = 10% or < 10%), but with much more favorable numbers needed to treat in those with CAC > 100 or when CAC is 1–100 if 10-year CHD risk is > 10%.

## Conclusions

Since the early findings of CAC as an indicator of intimal coronary atherosclerosis decades ago, a wealth of observational studies have documented the strong relation of CAC to cardiovascular outcomes, which is consistent regardless of sex or race/ethnicity. CAC has been shown to improve risk reclassification beyond global risk assessment more than any other measure of subclinical atherosclerosis. Moreover, the progression of CAC indicates greater future risk of cardiovascular outcomes. The presence and quantity of CAC appear to motivate initiation of preventive therapies and healthful behaviors; however, the role of CAC scanning for improving cardiovascular outcomes is not firmly established and awaiting the results of a major clinical trial. The ACC/AHA/Multisociety Cholesterol Management guideline, however, does incorporate CAC assessment into the treatment decision for consideration of statin therapy.
